# Morphology and cardiac physiology are differentially affected by temperature in developing larvae of the marine fish mahi-mahi (*Coryphaena hippurus*)

**DOI:** 10.1242/bio.025692

**Published:** 2017-04-21

**Authors:** Prescilla Perrichon, Christina Pasparakis, Edward M. Mager, John D. Stieglitz, Daniel D. Benetti, Martin Grosell, Warren W. Burggren

**Affiliations:** 1 University of North Texas, Department of Biological Sciences, Denton, TX 76203, USA; 2 Division of Marine Biology and Ecology, University of Miami, Rosenstiel School of Marine and Atmospheric Science, Miami, FL 33149, USA

**Keywords:** Mahi-mahi, Development, Heart rate, Stroke volume, Cardiac output, *Q*_10_

## Abstract

Cardiovascular performance is altered by temperature in larval fishes, but how acute versus chronic temperature exposures independently affect cardiac morphology and physiology in the growing larva is poorly understood. Consequently, we investigated the influence of water temperature on cardiac plasticity in developing mahi-mahi. Morphological (e.g. standard length, heart angle) and physiological cardiac variables (e.g. heart rate *f*_H_, stroke volume, cardiac output) were recorded under two conditions by imaging: (i) under acute temperature exposure where embryos were reared at 25°C up to 128 h post-fertilization (hpf) and then acutely exposed to 25 (rearing temperature), 27 and 30°C; and (ii) at two rearing (chronic) temperatures of 26 and 30°C and performed at 32 and 56 hpf. Chronic elevated temperature improved developmental time in mahi-mahi. Heart rates were 1.2–1.4-fold higher under exposure of elevated acute temperatures across development (*Q*_10_≥2.0). *Q*_10_ for heart rate in acute exposure was 1.8-fold higher compared to chronic exposure at 56 hpf. At same stage, stroke volume was temperature independent (*Q*_10_∼1.0). However, larvae displayed higher stroke volume later in stage. Cardiac output in developing mahi-mahi is mainly dictated by chronotropic rather than inotropic modulation, is differentially affected by temperature during development and is not linked to metabolic changes.

## Abstract (precis)

**Summary:** Acute and chronic temperature exposures affect differentially heart rate, stroke volume and cardiac output in mahi-mahi (*Coryphaena hippurus*) during early development.

## INTRODUCTION

Recruitment of fish populations is highly dependent upon environmental conditions encountered during early life-history stages (Houde, 1989; Rijnsdorp, 2009; Wexler et al., 2007). The sensitivity of embryos and larvae may be explained by their small size, incomplete morphological and physiological development, high dynamic metabolic rate, low energy reserves, small migration capacities (little or no swimming activity) and their heavy dependence on ambient environmental conditions (dissolved oxygen, temperature, pH, etc.) (Finn and Kapoor, 2008). This makes larval fishes more vulnerable to mortality during periods of adverse environmental conditions (food shortage, pollution, predation pressure, etc.) than the adult phenotype of the species (Rijnsdorp, 2009; Tyus, 2011). Ecosystem changes in response to climate change are mainly driven by the global warming trend (Perry et al., 2005; Rosenzweig et al., 2008; Walther et al., 2002). Developing fish, in particular, have a narrower thermal window than the later stages of their life history, making them particularly vulnerable to temperature changes (Pelster, 1999; Pörtner and Farrell, 2008; Pörtner et al., 2001; Rijnsdorp, 2009).

Temperature influences and constraints on amphibian and fish development primarily involve change in size and duration of the period when larvae are susceptible to modification of the normal ontogeny dynamic (Atkinson, 1995; Elliott and Elliott, 2010; Green and Fisher, 2004; Pelster, 1999; Pörtner et al., 2001; Rijnsdorp, 2009). The cardiovascular system is the first organ system to function in vertebrate embryos, so slight variations in cardiovascular function at these early stages could significantly affect subsequent development and/or species survival. For instance, temperature is a major modulator of intrinsic heart rate, typically having a *Q*_10_ value of ∼2.0 (Barrionuevo and Burggren, 1999; Farrell, 2009; Kopp et al., 2005; Schönweger et al., 2000). A rising temperature typically increases the oxygen demand and therefore requires some adjustments in cardiac activity (at least in adult stages) because of the respiratory and circulatory convection needed to deliver sufficient oxygen to the body tissues (Pelster, 1999). Consequently, the cardiac output (amount of blood pumped by the heart per minute) must be finely regulated.

Heart rate increases significantly with elevated rearing temperature in larvae of freshwater fish such as rainbow trout (*Oncorhynchus mykiss*) (Mirkovic and Rombough, 1998), zebrafish (*Danio rerio*) (Barrionuevo and Burggren, 1999) and the common minnow (*Phoxinus phoxinus*) (Schönweger et al., 2000). In the common minnow, these heart rate changes resulting from temperature variations increased during development as well. However, heart rate does not indicate all the physiological changes, because the chronotropic response was different to that for ventricular performance, where ventricular end-diastolic volume and stroke volume were higher at lower temperatures (15 and 17.5°C) and during initial cardiac activity in early development. Yet, overall cardiac output increased only at higher incubation temperatures and during later larval stages (e.g. swim bladder inflation stage) (Schönweger et al., 2000). A positive correlation exists between cardiac output and increasing incubation temperature, as well as with tissue mass, in advanced larval stages of rainbow trout (Mirkovic and Rombough, 1998)*.*

Adult fish typically exhibit changes in cardiac rate and contractility in response to environmental challenges like temperature change. However, changes that occur in cardiac performance are much smaller than concurrent changes in metabolism in larval fish (Pelster, 1999; Schönweger et al., 2000). Evidence clearly points to a link between metabolism and cardiac performance in adult vertebrates, and this link represents the main driver for adaptions and adjustments of cardiac activity in response to changing environmental conditions. However, it is unclear whether this relationship exists in embryonic and early larval stages of ectothermic vertebrates, in which the circulatory system does not initially play a primary role in oxygen delivery (Burggren, 2005, 2013; Cano-Martínez et al., 2007; Mirkovic and Rombough, 1998; Pelster and Burggren, 1996). In the natural environment, these physiological and morphological changes are influenced by temperature changes, which may cause significant fluctuations in productivity and distribution of fish populations, therefore leading to important ecological and evolutionary consequences (Pörtner, 2001; Pörtner et al., 2001).

Small temperature variation may have a greater impact on development of tropical fish than temperate fish, the latter subjected to a larger environmental temperature variation (Green and Fisher, 2004). Consequently, we elected to study thermal influences on cardiac physiology and morphology in the mahi-mahi (also known as the common dolphinfish), *Coryphaena hippurus*. Mahi-mahi is a migratory epipelagic fish species inhabiting tropical and subtropical waters (Beardsley, 1967; Gibbs and Collette, 1959; Palko et al., 1982). Mahi-mahi provide important commercial and sports fisheries in the Gulf of Mexico and others areas where they are commonly found (Oxenford, 1999). Despite the ecological and economic importance of this species, mahi-mahi provide some relevant benefits as a fish model for physiological and environmental studies. Most important is the very short embryonic development compared to zebrafish model [hatching between 36-45 h post-fertilization (hpf) at water temperatures of 25-28°C] and remain relatively transparent until 128 hpf.

We hypothesized that mahi-mahi, as a very rapidly growing and high performance fish, would be particularly vulnerable in early life stages to temperature fluctuations within their normal range and could increase exposure to the high risk pelagic environment. Thermally related physiological responses in larval mahi-mahi were measured under two rearing temperatures (26 and 30°C, defined in this study as chronic temperature exposure) and acutely exposed to three temperatures (25, 27 and 30°C). Worldwide temperature distribution of mahi-mahi varies between 25 and 31°C, so measurements were performed within this thermal range (Gibbs and Collette, 1959; Oxenford, 1999; Palko et al., 1982).

## RESULTS

### Temperature influence on embryo-larval morphology

**Fig. 1.**
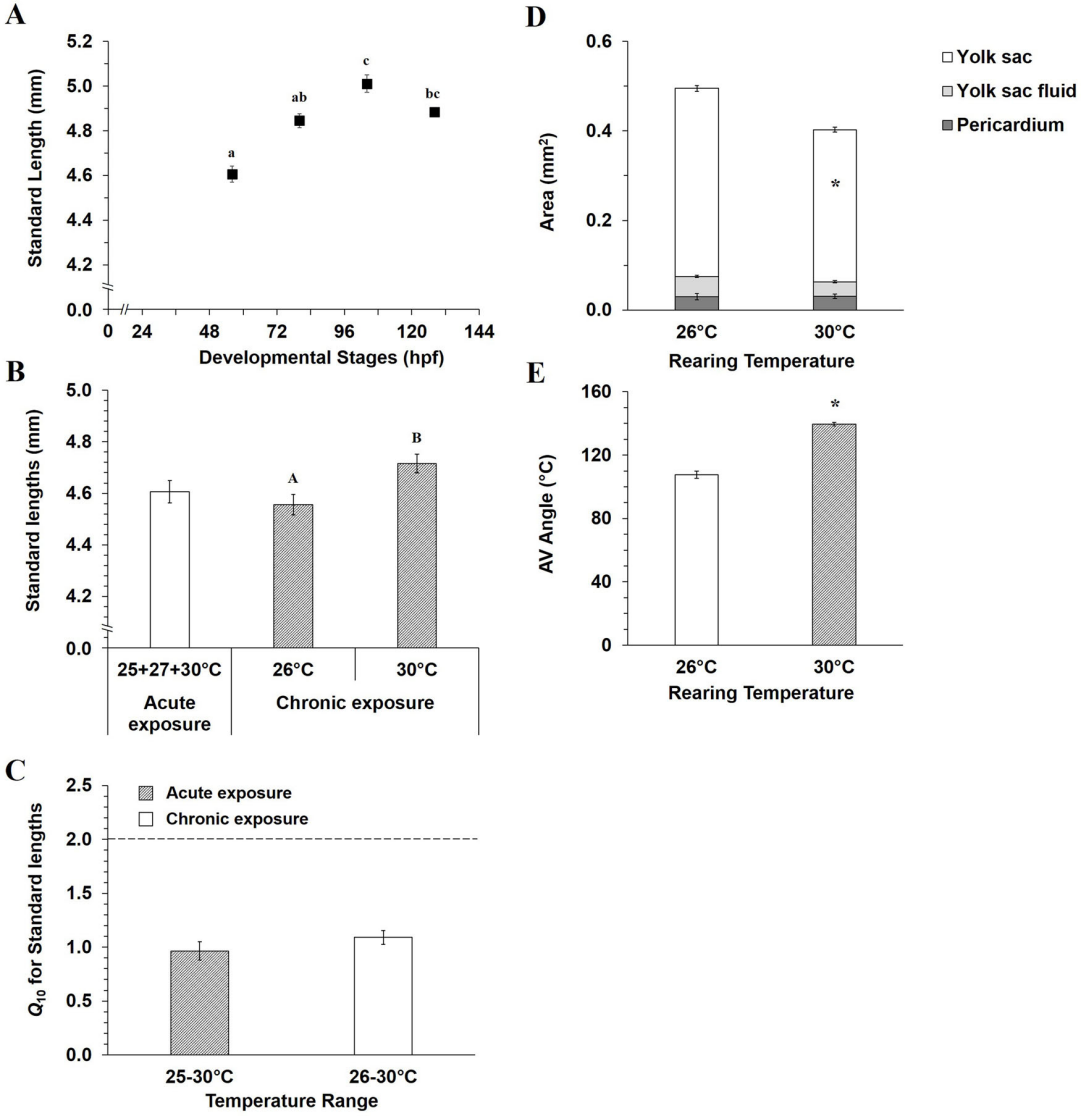
**Temperature influence on whole body morphology in early life stages of mahi-mahi.** (A) Standard length as function of development in mahi-mahi raised at 25°C and then acutely exposed during 20 s to 25, 27 and 30°C. Standard body lengths (B) and related *Q*_10_ (C) in hatched larvae (56 hpf) acutely and chronically temperature exposed. Pericardio-vitelline areas (D) and the atrio-ventricular angle (E) in 56 hpf larvae chronically temperature exposed. Data are presented as mean±s.e.m. *N*=30. *N*=42 and 56 in chronic assay for 26 and 30°C, respectively. Small and capital letters denote significant differences for acute and chronic temperature assays, respectively (ANOVA and Student's *t*-test, *P*<0.05). Asterisks in D and E indicate differences between both temperature conditions (26 and 30°C). No significant differences were found in pericardium and yolk sac fluid areas between both rearing temperatures (Student's *t*-test, *P*>0.05).

Standard body length increased with development (Fig. 1A, ANOVA, *P*=0.005). No differences in body length at any developmental stage occurred in hatched larvae raised at 25°C and acutely exposed to 25, 27 and 30°C. Thus data have been averaged (Fig. 1A, two-way ANOVA, *P*>0.05). However, mahi-mahi chronically raised at 30°C (4.72±0.04 mm) were longer than those chronically raised at 26°C (4.56±0.04 mm) (Fig. 1B, *P*=0.02). Despite these body length changes in 56 hpf larvae, *Q*_10_ values of ∼1.0 suggest no impact due to length of temperature exposure (Fig. 1C).

Larvae at 56 hpf chronically raised at 30°C displayed other morphological changes. No difference in pericardial area or yolk sac fluid accumulation were evident between two chronic rearing temperatures (Fig. 1D, *P*>0.05). Yolk sac resorption occurred earlier in the larvae reared at 30°C, and yolk sac area was reduced (0.34±0.005 mm^2^) compared to larvae reared at 26°C (0.42±0.007 mm^2^) (Fig. 1D, *P*<0.001). Additionally, specimens chronically raised at 30°C displayed an increased AV angle (140±1°) compared to larvae raised at 26°C (108±2°) (Fig. 1E, *P*<0.001).

### Development and cardiac function

**Fig. 2.**
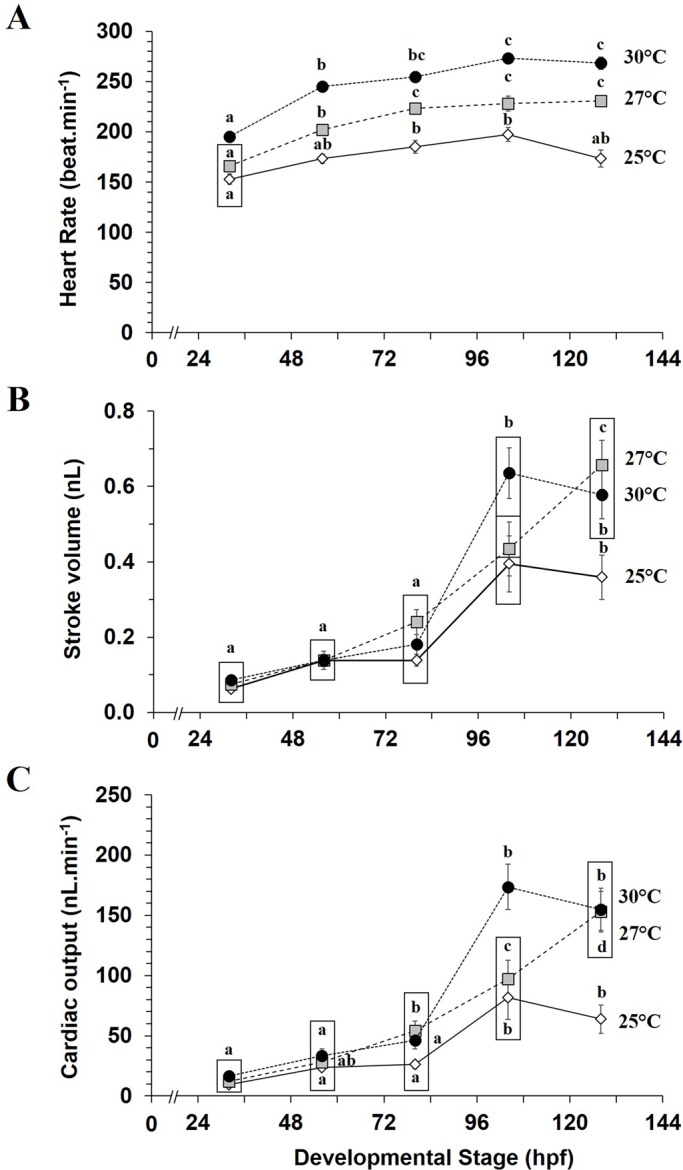
**Acute temperature influence on cardiac function of mahi-mahi during early development**. (A) Heart rate, (B) stroke volume and (C) cardiac output measured in mahi-mahi raised at 25°C and acutely exposed to 25, 27 and 30°C. Data are mean±s.e.m. *N*=10-11 for each plotted developmental stage per temperature. Boxes surround statistically identical values at the same measurement time. Letters denote significant differences between different developmental time at same temperature (two-way ANOVA, *P*<0.05).

Heart rate at a standard temperature of 25°C (Fig. 2A) increased with normal development (*P*<0.05), from 153±5 beats min^−1^ at 32 hpf to 185±6 beats min^−1^ at 80 hpf and then become stable until 128 hpf (198±7 and 168±10 beats min^−1^ at 104 and 128 hpf, respectively). Stroke volume and cardiac output showed similar variation patterns, both increased during development (Fig. 2B,C). Stable values were observed between 32 and 80 hpf, and then both significantly increased and reached a plateau around 80 hpf. When reared at 25°C, larval stroke volume and cardiac output increased by 2.8- and 3.1-fold between 80 and 104 hpf, respectively (*V*_H_=0.14±0.02 to 0.40±0.07 nl; *Q̇*=26.2±3.4 to 81.7±18.0 nl min^−1^).

### Temperature and cardiac function

Heart rate in embryos and larvae reared at 25°C then acutely exposed to 27 and 30°C were considerably higher across development (Fig. 2A). For example, heart rate in 56 hpf larvae reared at 25°C and then acutely exposed to 27 and 30°C were 1.2- and 1.4-fold higher, respectively, than heart rate measured in larvae at 25°C. Variation pattern in heart rate across development was similar at 27 and 30°C with an increase of heart rate from 32 to 56-80 hpf and then become constant later in stage. Stroke volume (Fig. 2B) and cardiac output (Fig. 2C) were 1.6- and 2.1-fold higher, respectively, at 104 hpf in larvae reared at 25°C and acutely exposed to 30°C (*P*<0.05). Both 27 and 30°C acute temperatures significantly increased stroke volume (1.8-1.6-fold) and cardiac output (2.4-fold) at 128 hpf (*P*<0.05).

**Fig. 3.**
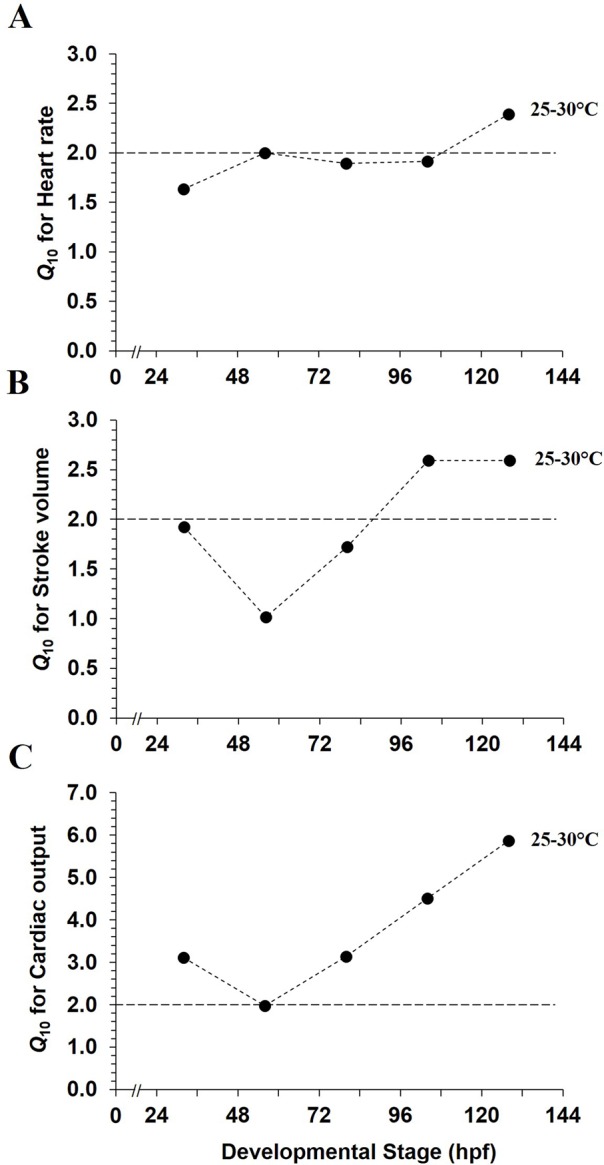
**Acute temperature coefficients for cardiac variables as functions of development in mahi-mahi**. *Q*_10_ values for (A) heart rate, (B) stroke volume and (C) cardiac output represent the temperature variation from 25 to 30°C. Each plotted point is a value calculated from the mean values extracted from previous Fig. 2 at each tested temperature. Dotted line is the *Q*_10_ value of 2.0.

*Q*_10_ values from 25 to 30°C for cardiac variables as a function of development are presented in Fig. 3. *Q*_10_ for heart rate increased from 1.6 to 2.0 after hatching (Fig. 3A), was constant until 1.4 hpf and then increased to 2.4 at 128 hpf. *Q*_10_ values for stroke volume (Fig. 3B) decreased from 1.9 to 1.0 between 32 and 56 hpf and then greatly increased until a plateau was reached at 104 and 128 hpf with *Q*_10_ of 2.6. Over a similar temperature variation, *Q*_10_ pattern for cardiac output (Fig. 3C) was above 2.0, with a decrease from 3.1 to 2.0 between 32 and 56 hpf. *Q*_10_ then greatly raised thereafter reaching a *Q*_10_ of 5.9 at 128 hpf.

**Fig. 4.**
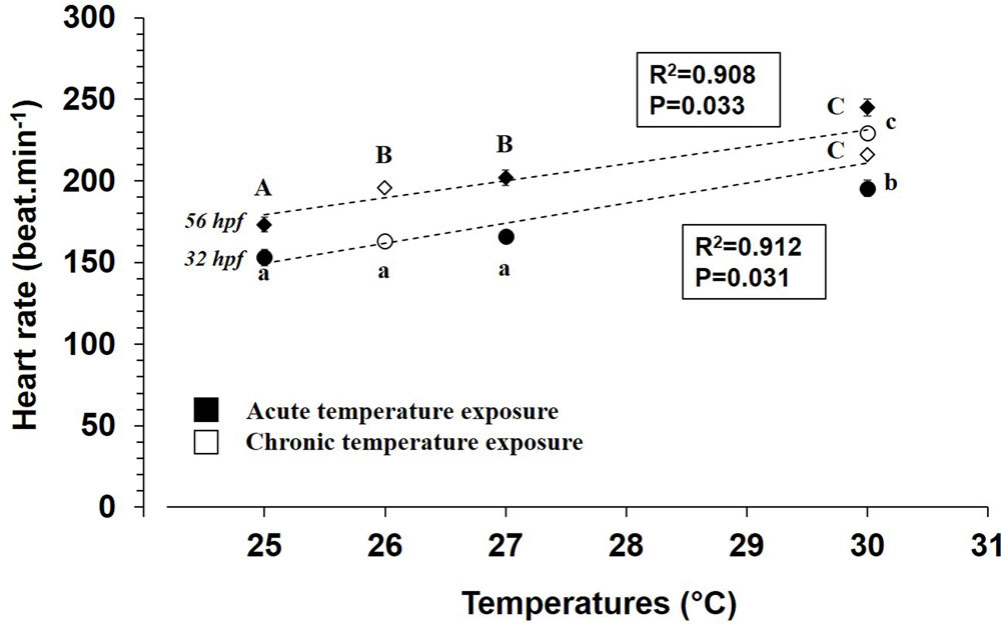
**Temperature influences on heart rate in embryos (32 hpf) and hatched larvae (56 hpf) of mahi-mahi during acute and chronic temperature exposures.** Data are mean±s.e.m. *N*=10-11 in acute assays at 32 and 56 hpf. At 32 hpf, *N*=51 at 26°C and 35 at 30°C. At 56 hpf, *N*=99 at 26°C and 68 at 30°C in chronic temperature assays. Small and capital letters indicate significant differences at 32 hpf and 56 hpf stages, respectively (ANOVA, *P*<0.05).

A linear relationship between heart rate and increasing temperature was observed during acute and chronic assays, with a relatively high correlation coefficient at 32 hpf (R^2^=0.912, *P*=0.031) and 56 hpf (R^2^=0.908, *P*=0.033) (Fig. 4).

**Fig. 5.**
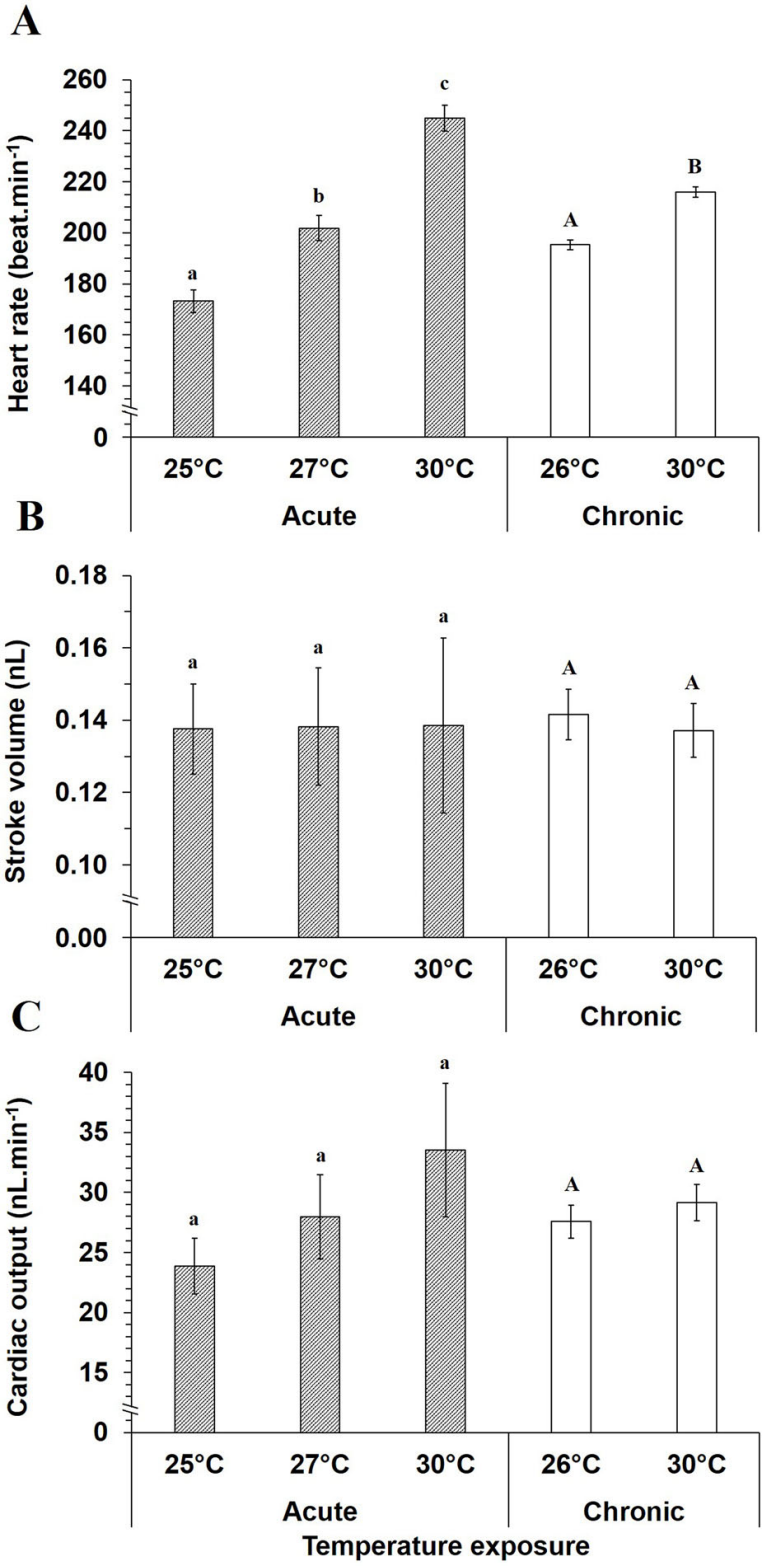
**Acute and chronic temperature influences on cardiac function in 56 hpf mahi-mahi**. Data are mean±s.e.m. for (A) heart rate, (B) stroke volume and (C) cardiac output. *N*=10-11 for each temperature in acute assay. *N*=42 and 56 in chronic assay for 26 and 30°C, respectively. Small and capital letters denote significant differences in acute and chronic assays, respectively (ANOVA and Student's *t*-test, *P*<0.05). No temperature effect was shown on stroke volume and cardiac output irrespective of exposure protocol (*P*>0.05).

Specifically, at 56 hpf, heart rate of mahi-mahi larvae were elevated 1.4- and 1.1-fold with increasing temperatures during acute and chronic temperature exposures, respectively (Fig. 5A, *P*<0.05). Stroke volume and cardiac output were not significantly affected by increasing temperature at 56 hpf, irrespective of applied thermal challenge (Fig. 5B, *P*>0.05). While no statistical difference occurred in cardiac output during acute temperature exposure, values tended to increase with elevated temperature (Fig. 5C).

**Fig. 6.**
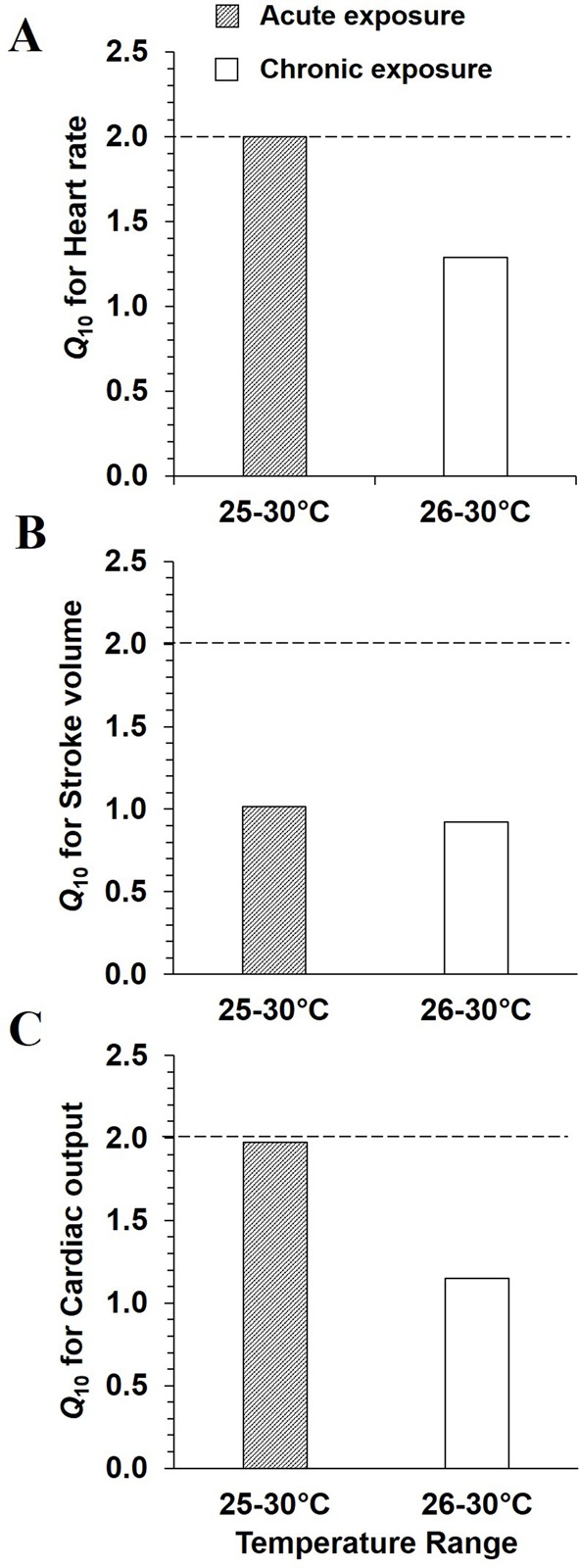
**Acute and chronic temperature coefficients for cardiac variables in 56 hpf mahi-mahi**. *Q*_10_ values for (A) heart rate, (B) stroke volume and (C) cardiac output, represent two temperature variations: 25-30°C and 26-30°C in acute and chronic assays, respectively. Each plotted point is a value calculated from the mean values at each tested-temperature. Dotted line is the *Q*_10_ value of 2.0.

*Q*_10_ values for heart rate in 56 hpf hatched larvae illustrated temperature dependence in acute exposure with *Q*_10_ value of 2.4 (Fig. 6A). *Q*_10_ is 1.8-fold lower for heart rate in chronic temperature exposure. For stroke volume, *Q*_10_ was maintained at ∼1.0 at 56 hpf (Fig. 6B). Interestingly, despite no statistical changes in cardiac output whatever the acute or chronic conditions, *Q*_10_ for this variable increased to around 2.0 when measured acutely over the temperature range of 25-30°C (Fig. 6C), indicating a trend to increase. However, *Q*_10_ was 1.1 when comparing cardiac output over the range of the two chronic rearing temperatures, 26-30°C.

## DISCUSSION

The development of cardiac functional and morphological parameters has been explored in numerous freshwater and saltwater fish species (Bagatto, 2005; Bagatto and Burggren, 2006; Burggren and Bagatto, 2008; Burggren and Blank, 2009; Farrell et al., 2009; Incardona et al., 2014; Jacob et al., 2002; Pelster, 1999). The rationale driving this research has been thoroughly outlined with prominent arguments based on the importance of the heart and the impact the developmental environment can have on morphological and function phenotypes. Therefore, to further document this critical process, we explored the influence of temperature on development of cardiac form and function in embryonic and larval stages of mahi-mahi. To our knowledge, the literature exploring the physiological capabilities in early development of mahi-mahi due to environmental changes (such as temperature) is very limited. The present study is the first to report acute and chronic temperature effects on the development and cardiac filling changes (stroke volume and cardiac output) in the embryonic stages of mahi-mahi.

### Challenges to cardiovascular measurements in mahi-mahi

Although morphological changes during development can be readily monitored, assessment of cardiac function during early heart development can be challenging. First, cardiogenesis progresses rapidly (hours to a day) in many warm water fish species. In mahi-mahi, at 26°C primitive heart (precursors) appears by 18-19 hpf, with the onset of heart beat by 22 hpf. By 50-56 hpf, the sinus venosus and bulbus cordis start to be differentiated and the heart starts to loop to the lateral side, as it begins to assume its adult configuration over 80 hpf with formation of both valves (P.P., M.G., W.W.B., unpublished data).

A second challenge to cardiovascular measurements is imposed by the small size of larval mahi-mahi. The use of digital imaging methods in embryos and larvae, while feasible, is labor-intensive, which can limit sample size. However, digital imaging does allow observation of dynamic process of cardiovascular parameters *in vivo*. Furthermore, the accuracy and reliability of these techniques has been well established in larvae of freshwater fish and amphibians (Bagatto and Burggren, 2006; Hou and Burggren, 1995; Kopp et al., 2014; Mirkovic and Rombough, 1998; Schönweger et al., 2000).

Finally, a third challenge is modeling the shape of the ventricle in a manner that most accurately reflects this complex structure (P.P., M.G., W.W.B., unpublished data). During the early developmental phase, the heart of mahi-mahi is somewhat irregularly shaped. As noted in the Materials and Methods, the ventricle is relatively elongate at 50-56 h, but quickly assumes a shape more like a prolate spheroid with further development (P.P., M.G., W.W.B., unpublished data). In this study, we have modeled all stages on the commonly used prolate spheroid model, allowing us to focus on relative changes between stages and temperatures for better comparison with existing data in literature. However, future studies will gain even greater accuracy by using stage-specific modeling of ventricular shape.

### Standard cardiac development at rearing temperature of 25°C

Heart rate in developing mahi-mahi initially increases from 32 to 80 hpf and becomes stable thereafter. These results are qualitatively similar to other fish species, such as zebrafish (Bagatto and Burggren, 2006; Barrionuevo and Burggren, 1999; Denvir et al., 2008; Jacob et al., 2002), common minnow (Schönweger et al., 2000), rainbow trout (Mirkovic and Rombough, 1998), several species of tuna (Pacific bluefin tuna *Thunnus orientalis*, Atlantic bluefin tuna *Thunnus thynnus*, and yellowfin tuna *Thunnus albacares*) (Clark et al., 2013; Incardona et al., 2014), and the greater amberjack (*Seriola dumerili*) (Incardona et al., 2014). At a standard rearing temperature of 25°C, stroke volume and cardiac output variations tend to increase across development. Until 80 hpf, they are relatively constant and then during mouth opening and resorption of yolk sac stage (104 hpf), stroke volume and cardiac output increased threefold [*V*_H(104hpf)_=0.40±0.07 nl and *Q̇*_(104hpf)_=81.7±18.0 nl min^−1^] compared to 80 hpf. These values were then constant at later stages.

Heart rate is considered a highly accurate proxy used in the literature regarding modification of cardiac performance in fishes (Clark et al., 2013; Edmunds et al., 2015; Incardona et al., 2009, 2014; Mager et al., 2014; Perrichon et al., 2016). This variable provides insight into, but not a comprehensive picture of, the dynamics of blood pumping. Stroke volume and cardiac output should be included as new functional variables when evaluating cardiac function in fish early life stages exposed to environmental challenges. These variables could provide an explicit physiological expression of the environmental impact while acting as an estimate and predictor of impairment and risk arising from challenging natural or anthropogenic conditions.

Little is known about cardiac performance in developing marine fish and how it is impacted by environmental factors such as temperature. To our knowledge, this is the first study reporting quantitative data of stroke volume and cardiac output in developing pelagic marine fish. The developing coastal Gulf of Mexico fish species, the red drum (*Sciaenops ocellatus*) (Khursigara et al., 2017), at similar stage (56 hpf) and temperature (25°C), have stroke volumes and cardiac outputs that are 2.8- and 3.4-fold lower respectively, compared to mahi-mahi. Previous studies using similar imaging method measurement on developing zebrafish, common minnow or rainbow trout have reported accurate measurement of stroke volume and cardiac output (Bagatto and Burggren, 2006; Kopp et al., 2005; Mirkovic and Rombough, 1998; P.P., M.G., W.W.B., unpublished data; Schönweger et al., 2000). Cardiac output measurements are mass specific, therefore a direct comparison of data between species is not possible considering organisms weighing less than 1 mg (Mirkovic and Rombough, 1998; Schönweger et al., 2000). However, even given existing differences in life cycle and species, our data compare favorably to the limited literature values.

### Enhancement of early larval development by chronic warming temperature

Not surprisingly, an acute increase in temperature in short duration above the standard rearing temperature of 25°C did not affect the body length of larvae (*Q*_10_≤1.0). However, larvae chronically exposed to 30°C from hatching displayed greater body length than those raised at 26°C. These changes in size were accompanied by a reduction of the yolk sac volume, which likely indicates a faster absorption of vitelline reserve associated with the presumably elevated metabolic rate and more rapid advancement through successive larval stages (Pasparakis et al., 2016). Furthermore, chronic elevated temperature accelerated the lateral repositioning of cardiac chambers in hatched larvae of mahi-mahi, as quantified by an increase in the atrio-ventricular angle (Fig. 1). These changes in cardiac angle may be associated with some adjustments in preload, afterload and contractility, all factors modulating stroke volume and therefore cardiac output (Edmunds et al., 2015; Kalogirou et al., 2014). Indeed, even if no evidence of direct functional disruption is shown, and it might simply be due to advanced development, an increase of this angle might result in cardiac function depression in aquaculture related to domestication, or again after crude oil exposure, leading to changes in general heart tube patterning (Edmunds et al., 2015; Hicken et al., 2011). Thus, our findings regarding temperature-induced body length changes for mahi-mahi are similar to those previously documented (Green and Fisher, 2004; Wexler et al., 2011).

Embryonic development is highly energetically costly (Rombough, 2006, 2011). Increasing temperature exacerbates the impact by increasing the energetic extraction from finite yolk reserves of the developing fish. Tropical fishes develop faster, hatch earlier and grow up more rapidly than more temperate fishes (Val et al., 2005). As a consequence, the vitelline reserve is rapidly exhausted, which necessitates a quick transfer to reliance upon exogenous food sources (Kim et al., 2015; Yúfera and Darias, 2007). Rapid depletion of the yolk sac with increasing temperature has been documented in at least four marine species: porgy (*Acanthopagrus schlegeli*), Japanese anchovy (*Engraulis japonica*), red sea bream (*Pagrus major*) and Japanese flounder (*Paralichthys olivaceus*) (Fukuhara, 1990). In these species, earlier yolk depletion is associated with faster development and in the accelerated development of swimming ability, which is temperature-dependent (Fukuhara, 1990). In fish, exposure temperatures near the lower and upper tolerance limits may delay hatching and induce morphological and cardiovascular impairments (Burggren and Bagatto, 2008; Clark et al., 2013; Pörtner and Farrell, 2008; Schirone and Gross, 1968). In the present study, increasing temperature within a range of optimal water temperature tolerance enhances the development time of mahi and thereby increases larval size. As previously suggested, faster development may help to quickly develop swimming ability, which might be beneficial for migration, seeking food or avoiding predators (Blaxter, 1991; Houde, 1989). In contrast, acceleration of development at the time of yolk sac absorption might be harmful and limiting the optimal growth of the larvae. The yolk sac is the sole source of nutrients in the embryo, so an excessively rapid depletion might reduce the nutritional state of organism and favor developmental abnormalities. This could reduce the first feeding success, thereby increasing the chance of mortality in the pelagic environment.

### Temperature as a positive chronotrope

Warmer temperatures strongly affected heart rate during early development of mahi-mahi. Analysis of *Q*_10_ patterns revealed a great sensitivity of heart rate to acute increases in temperature between 56 and 128 hpf, which matches *Q*_10_ values ≥2.0 (1.9 to 2.4) over this developmental range. This suggests that temperature influences cardiac pacemaker tissue function. In chronic exposure, the *Q*_10_ value of 1.3 indicates a slight influence of elevated temperature in hatched larvae compared to those acutely exposed. Temperature triggers change in cardiac physiological performance in larval fish, with published *Q*_10_ values for heart rate of ∼2.0 in both isolated hearts and intact fish (Burggren et al., 2016; Farrell, 2009; Gamperl and Farrell, 2004; Graham and Farrell, 1989). Patterns of heart rate due to increasing temperature similar to mahi-mahi occur in freshwater fish, such as the common minnow (Schönweger et al., 2000) and zebrafish (Barrionuevo and Burggren, 1999), resulting in *Q*_10_ values of 1.8 and from 1.2 to 2.5 over the temperature ranges of 15-25°C and 25-31°C, respectively. In cold water species, *Q*_10_ values of 2.2-2.4 and 2.6 were also reported in embryos of the speckled trout (*Salvelinus fontinalis*) and Atlantic salmon (*Salmo salar*), respectively (Fisher, 1942; Klinkhardt et al., 1987; Pelster, 1999).

### Temperature independence of stroke volume and cardiac output

Stroke volume and cardiac output exhibited similar patterns during the earliest development (up to 128 hpf) of mahi-mahi. At all three acute temperatures (25, 27 and 30°C), increased heart rate was not directly associated with any change in stroke volume and cardiac output from 32 to 80 hpf of development. However, from 104 hpf of development, hatched larvae showed a significant elevation in both stroke volume and cardiac output whereas heart rate remain constant. Regarding the acute temperature of 27°C, embryos and newly hatched larvae (56 hpf) both displayed significantly higher heart rate. At the same temperature, cardiac output elevation was related to increased heart rate from 80 hpf and related to greater stroke volume from 104 hpf.

Cardiac output increased linearly during development, directly linked with similar increase in stroke volume, whereas heart rate remain constant. Increased stroke volume and cardiac output are likely related to greater development and increased body size. Cardiac volumes are even more important at the warmest exposure temperatures at 104 hpf and 128 hpf (stroke volume and cardiac output are 1.6- and 2.1-2.4-fold higher, between 25 and 30°C, respectively). *Q*_10_ values corroborate this influence, with *Q*_10_ for stroke volume and cardiac output being 2.6 and 4.5-5.9, respectively, in 104 hpf mahi-mahi larvae. Considering larvae at 56 hpf, both acute and chronic increases in temperature created a similar range of variation in cardiac performance. Yet, cardiac output was not significantly affected by temperature at this developmental stage, but tended to increase. This slight increase (trend) is due to the product of heart rate and stroke volume, with heart rate playing a greater role in cardiac output regulation. Collectively, this trend and calculated *Q*_10_ values lead us to conclude that cardiac output is influenced to a greater extent later in larval development. Our results also highlight that cardiac output in embryonic and newly hatched larva might be mainly dictated by chronotropic rather than inotropic modulation. Similarly, in the larval common minnow, incubation temperature did not create any change in stroke volume and cardiac output (*Q*_10_=1.40) (Schönweger et al., 2000). In the adult isolated trout heart, no major changes in stroke volume result from increasing chronic temperature (*Q*_10_=1.3-1.4), while heart rate and cardiac output were significantly higher (Graham and Farrell, 1989). The equal stroke volumes observed in embryonic and newly hatched larval mahi-mahi (32 hpf to 80 hpf) under acute and chronic temperature conditions might result from extrinsic compensatory mechanisms.

### Dissociation of cardiac performance and oxygen consumption

A marked increase in oxygen consumption occurs as a consequence of increasing chronic rearing temperature from 26 to 30°C in mahi-mahi embryos and newly-hatched larvae (Pasparakis et al., 2016). A clear increase in energy demand was particularly evident when embryos approached hatching and especially so at higher temperatures. Chronic *Q*_10_ for oxygen consumption calculated on published data for mahi-mahi (Pasparakis et al., 2016) yielded a *Q*_10_ value of 3.6 in newly hatched larvae (56 hpf). Our study has clearly demonstrated a temperature influence on early rhythmicity of cardiac function in developing mahi-mahi under acute temperature change or chronic rearing conditions. Increasing heart rate in developing mahi-mahi coincides with an increase of oxygen consumption, but cardiac output seems to be independent of oxygen consumption at this developmental stage. Regarding the associated *Q*_10_, the influence of temperature appears greater in energy metabolism (oxygen consumption) than in heart rate at similar developmental stages. These differences in *Q*_10_ and the absence of correlation between cardiac output and metabolic rate are not especially surprising. Indeed, while the relation between metabolic demand of tissues and cardiac activity is well established in adult fish, no apparent link exists between these two variables in early life stages. Diffusion of oxygen transport through the skin and tissues likely suffices to supply oxygen to tissues making cardiac regulation independent of the circulatory system in larval fish (Burggren, 2005, 2013; Farrell et al., 2009; Mirkovic and Rombough, 1998; Pelster and Burggren, 1996; Schönweger et al., 2000).

### Conclusions and further directions

From an eco-physiological point of view, this study highlights the importance of measuring a range of physiologically relevant traits to characterize their relative condition during early development due to environmental change, and then understanding how these variations will influence later life. While heart rate has been used as an indicator of larval condition and health in the past, our data indicate that heart rate alone does not tell the full story of cardiovascular performance. We hypothesized that rapidly growing, high performing mahi-mahi would be particularly vulnerable as larvae to temperature fluctuations within their normal range. Yet, this hypothesis was only partially supported, as important cardiac variables showed small to no temperature dependency. Further studies should be undertaken to assess the cardiorespiratory capacity of developing fish and define the connection period between cardiac performance and metabolic supply. Furthermore, while considerable information is available about general aspects of development, the underlying mechanisms of cardiac physiological responses to temperature are poorly understood in early life stages and warrant further investigation.

## MATERIALS AND METHODS

### Maintenance and egg production of mahi-mahi

Mahi-mahi broodstock were captured off the coast of Miami, FL, USA using hook and line angling techniques. The fish were subsequently transferred to the University of Miami Experimental Hatchery (UMEH), where they were acclimated in 80 m^3^ fiberglass maturation tanks equipped with re-circulated and temperature controlled water (∼26°C). All embryos used in the experiments described herein were collected within 8 h following a volitional (non-induced) spawn using standard UMEH methods (Stieglitz et al., 2012). A prophylactic formalin treatment (37% formaldehyde solution at 100 μl l^−1^ for 1 h) was administered to the embryos, followed by 30 min of flushing with a minimum of 300% water volume in the treatment vessel using filtered, UV-sterilized seawater. A small sample of embryos was then collected from each spawn to microscopically assess fertilization rate and embryo quality. Spawns demonstrating low fertilization rate (<85%) or frequent developmental abnormalities (>5% of individuals) were not used.

All handling and use of animals in the present study were in compliance with the Institutional Animal Care and Use Committee (IACUC) of the University of Miami.

### Experimental protocols

Morphological and physiological data were acquired under two conditions: acute and a chronic temperature exposure. During acute exposures, morphological and functional variables were measured in a range of developmental stages of mahi-mahi that were initially reared at 25°C through 128 hpf and then acutely exposed (20 s) to 25 (rearing temperature), 27 or 30°C. For chronic rearing exposures, measurements were made at either 26 or 30°C, at only two developmental points, 32 and 56 hpf. Table 1 summarizes the variables measured during both acute and chronic temperature assays. Embryo and larvae developmental stages are expressed in hpf. The rearing temperatures of 25 and 26°C were chosen for the acute and chronic experiments, respectively, to match the temperatures at which the eggs were collected from the broodstock tanks.

**Table 1.**
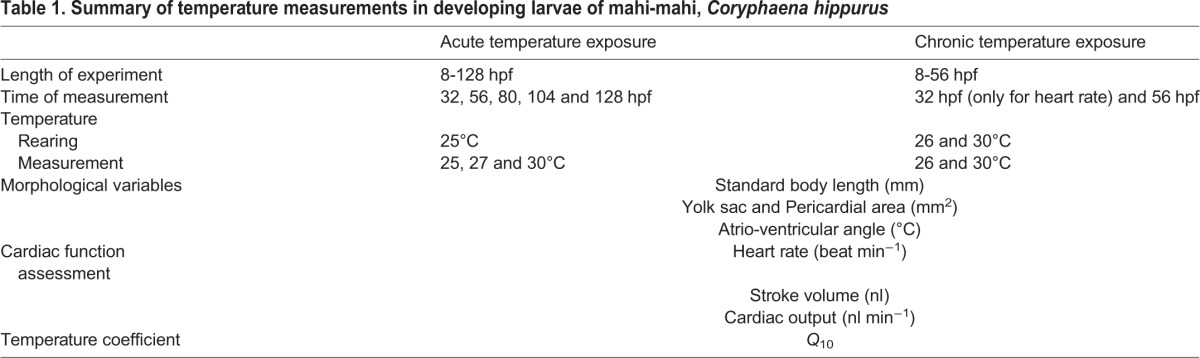
**Summary of temperature measurements in developing larvae of mahi-mahi, *Coryphaena hippurus***

### Data acquisition

#### Image and video capturing

Unanaesthetized embryos and larvae were individually immobilized in UV-sterilized seawater containing 2% methylcellulose (to increase viscosity) in a Petri dish. Individuals were positioned on a thermally regulated microscope stage (Brook Industries, Lake Villa, IL), oriented for ventral and left lateral views for embryos and larvae, respectively. Video images of the positioned individuals were captured using a Nikon SMZ800 stereomicroscope (objective lens 8× and 9.8×) connected to a Fire-i400 or Fire-i530c digital camera (Unibrain, San Ramon, CA). For morphological and functional assessment, 20-s-long live videos were digitized at 30 frames s^−1^ using PhotoBooth software (dslrBooth Lumasoft, East Brunswick, NJ). Images were calibrated using a stage micrometer.

#### Assessment of cardiac function

**Fig. 7.**
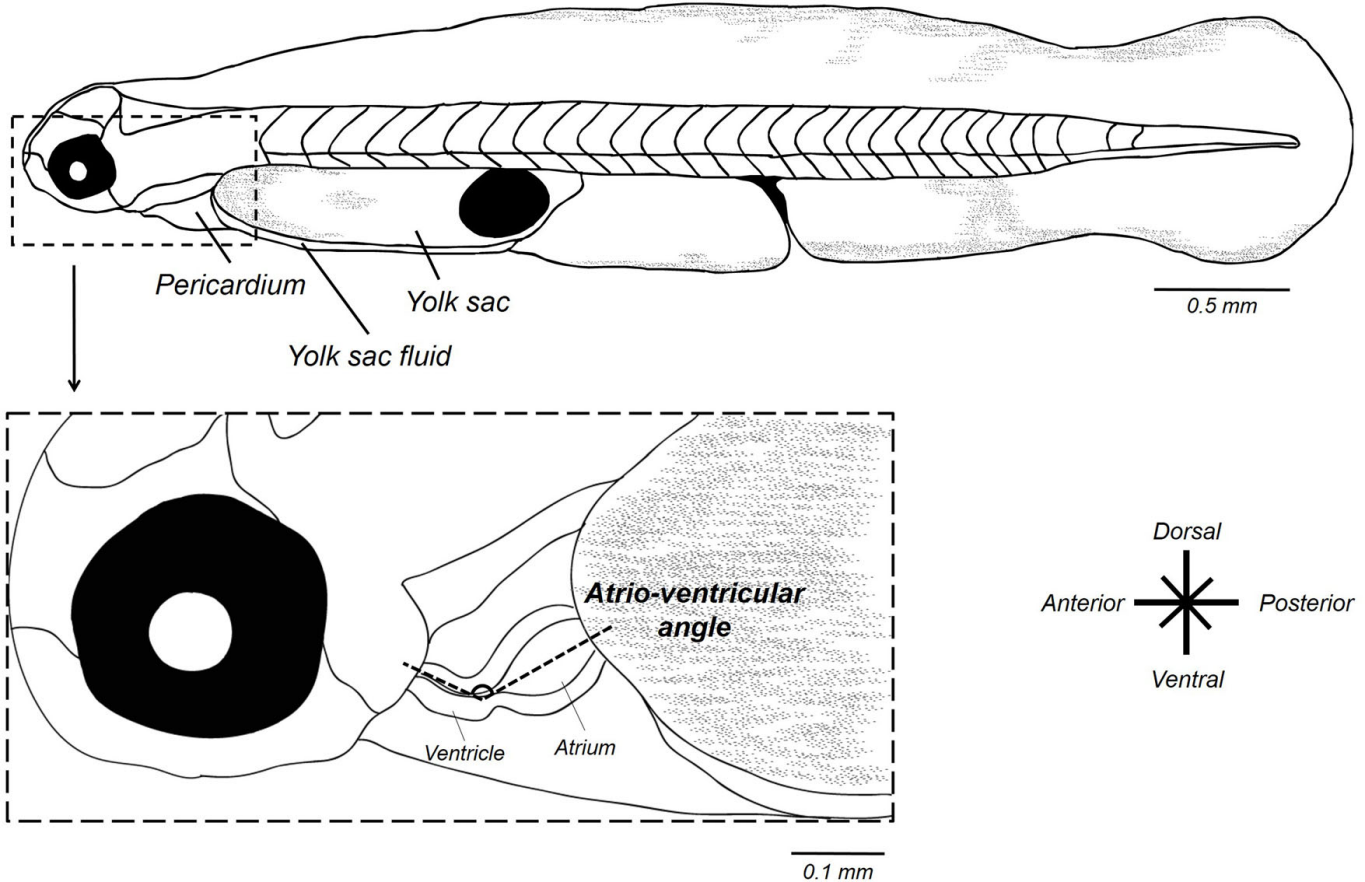
**Schematic representation of morphometric measurements of the heart in 56 hpf mahi-mahi larvae.**

Morphometric measurements were made using ImageJ software (Schneider et al., 2012; http://imagej.nih.gov/ij/) from acquired images as described above. Atrio-ventricular angle (AV) was measured with the ImageJ freehand tool, as two lines diverging from the center point of the maximally relaxed atrium and the maximally contracted ventricle (end-systolic) (Fig. 7). AV angle was used to estimate the looping of cardiac chambers (Edmunds et al., 2015). Angle measurements involved the average of three measurements from videos frames.

Heart rate (*f*_H_) and stroke volume (*V*_H_) variables were determined from video sequences of the ventricle in embryos and larvae and used for cardiac output calculation (*Q̇*). Heart rate (heartbeat min^−1^) was visually determined from slow speed videos. End-diastolic and end-systolic volumes of the ventricle were determined by outlining the ventricular perimeter (circumference) using the best fitting ellipse drawn with ImageJ tools (P.P., M.G., W.W.B., unpublished data). Major and minor axes were then determined, extracted and exported into a Microsoft excel worksheet.

An important potential limitation of this technique is that at approximately 50-56 h of development in mahi-mahi (the time of our first measurements) the anteriorly located ventricle is relatively elongate compared to the larger and nearly spherical posteriorly atrium (Edmunds et al., 2015; P.P., M.G., W. W. B., unpublished data). Within hours, however, the ventricle quickly assumes a more prolate spheroid shape as it grows rapidly to meet and then exceed the size of the atrium. Thus, it is easy for the observer to confuse atrium, ventricle and bulbus in early developmental stages, and especially during the early stages of coordinated heart chamber contraction. This makes quality of lighting and careful observation of moving erythrocytes and the timing of their movement between chambers of critical importance.

While recognizing the above limitations, as has been more fully explored (P.P., M.G., W.W.B., unpublished data), we were interested in cardiac output changes under thermal influence. Thus, stroke volume for all stages was calculated using the same formula – namely the formula for a prolate spheroid commonly used in previous studies on larval fishes and amphibians and embryonic birds (Bagatto and Burggren, 2006; Burggren and Fritsche, 1995; Burggren et al., 2004; Faber et al., 1974; Hou and Burggren, 1995; Keller et al., 1990; Kopp et al., 2005; Schönweger et al., 2000). This heart-shape model has been previously demonstrated to give the most accurate measurement of ventricular volumes (P.P., M.G., W.W.B., unpublished data):

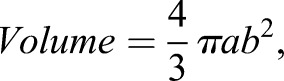


where *a* represents the major (longitudinal) semi-axis and *b* the minor (width) semi-axis.

Three systolic and diastolic events for each larvae were captured, analyzed and then averaged to minimize measurement error. Mean stroke volume (nl) was calculated as the difference between diastolic and systolic ventricular volumes. Ejection fraction (%) was calculated from stroke volume/diastolic volume. Finally, cardiac output (nl min^−1^) was calculated by multiplying the *f*_H_ by *V*_H_.

In mahi-mahi, hatching occurs between 41 and 45 hpf at 26°C. Standard length measurements were made in hatched larvae. Area of the yolk sac, internal yolk sac fluid and pericardial area were also determined with Image J.

#### Calculation of temperature sensitivities

The *Q*_10_ temperature coefficient represents a standardized measure of the change in rate (*R*) of a biological system when the temperature (*T*) is increased by 10°C. *Q*_10_ values were determined by the following equation:

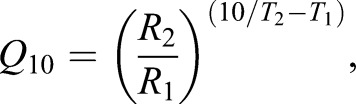


where *R*_1_ and *R*_2_ are the measured reactions rate at temperature *T*_1_ and *T*_2_ respectively (*T*_1_<*T*_2_). A *Q*_10_ value of 2.0 is typical of the normal rate of change of routine metabolism with temperature (Drost et al., 2014; Farrell et al., 2009; Fry, 1971; Randall et al., 2002).

### Statistical analysis

Statistical analyses were performed using Statistica 12 software package (Statsoft, Tulsa, OK, USA). We statistically evaluated morphological and functional variables with a one- and two-way ANOVA, followed by Tukey post hoc test for the acute temperature assays. In chronic rearing temperature assays, we used Student's *t-*tests to compare both exposure temperatures. Results are expressed as mean± s.e.m. Data from chronic temperature assays involved an average of six experiments in time. A significance level of 5% was used for all analyses.
